# Anthocyanins prevent the development and progression of urethane-induced lung cancer by regulating energy metabolism in mice

**DOI:** 10.29219/fnr.v68.10434

**Published:** 2024-04-17

**Authors:** Han Luo, Mengyuan Gao, Hong Lu, Qianyao Chen, Xuemei Lian

**Affiliations:** 1 Department of Nutrition and Food Hygiene, School of Public Health, Chongqing Medical University, Chongqing, P.R. China; 2 Center for Lipid Research, Key Laboratory of Molecular Biology for Infectious Diseases (Ministry of Education), Chongqing Medical University, Chongqing, P.R. China

**Keywords:** anthocyanin, lung cancer, energy metabolism

## Abstract

Anthocyanin (ACN) is a natural antioxidant with multiple biological activities, and the aim of this study was to evaluate the protective effect of ACN on the development and progression of lung cancer and to further explore its possible mechanism of action. In vivo, we fed C57BL/6J mice a 0.5%ACN diet or a control diet to observe their effects on the development and progression of urethane-induced lung cancer. In vitro, multiple lung cancer cell lines were used to investigate the effects of C3G on cell viability. The results showed a reduction in lung tumor burden and downregulation of oxidative phosphorylation and fatty acid degradation pathways in lung tissue of urethane-administrated ACN-fed mice compared with control diet-fed mice. In vitro, cyanidin-3-O-glucoside chloride (C3G) intervention treatment significantly inhibited proliferation and apoptosis of A549 cells. This process is likely due to the modulation of AMPK/mTOR signaling pathway by C3G to regulate cellular fatty acid metabolism and reduce intracellular lipid accumulation which affects the growth of lung cancer cells. These results suggest that ACN can inhibit the development and progression of urethane-induced lung tumors and alter the lipid metabolism of tumors in C57BL/6J mice.

## Popular scientific summary

- Anthocyanins inhibit the development and progression of urethane induced lung cancer in mice.
- Anthocyanins can affect energy metabolism in tumor cells.
- C3G inhibit proliferation, migration and apoptosis of lung cancer cells in vitro.
- C3G regulates cellular fatty acid metabolism and reduces the accumulation of Reference lipids by activating the AMPK/mTOR signaling pathway.

Lung cancer is a disease with high morbidity and mortality worldwide (second only to breast cancer in incidence), with a 5-year survival rate of only 24%; (1, 2). Although various methods such as surgery, chemotherapy, and radiotherapy are used to treat NSCLC and SCLC, the main treatment for lung cancer patients is chemotherapy (3, 4). Chemoprevention refers to the use of drugs or natural substances to delay or reverse the carcinogenic process and is one of several promising strategies to reduce cancer development (5). Many natural substances present in the human diet have been identified as potential chemopreventive and/or chemotherapeutic agents (6, 7). Most cancer incidence could be reduced by diets rich in fruits and vegetables (8–10). Therefore, the search for new natural agents and identification of new chemo preventive targets have become the focus of researches.

Anthocyanin (ACN) are a group of water-soluble natural pigments that belong to the flavonoid family and are present in a variety of vegetables and fruits. Because anthocyanins have certain structural properties, they are predominantly found in nature as glucosides (11). So far, more than 700 anthocyanin derivatives have been discovered, six of which are the main ones: cyanidin pigment, geranium pigment, peony pigment, delphinidin pigment, morning glory pigment, and mallow pigment (12, 13). Anthocyanins have a variety of biological activities, including anti-oxidation, anti-inflammation, antiaging, and prevention of coronary heart disease (14–16). Several studies have also demonstrated that anthocyanins can inhibit the development and progression of diverse malignancies, including breast, colon, liver, prostate, and skin cancers etc. (17–20). Even though it has been demonstrated that anthocyanins could prevent lung cancer in population studies (21), the mechanism by which anthocyanins inhibit lung cancer growth has not been thoroughly investigated.

Fatty acids are important energy sources and cellular structural components in most organisms. Fatty acid metabolism is essential for energy production and synthesis of new lipid metabolites (22). Previous studies have shown that upregulated fatty acid levels are associated with an increased risk of developing cancer, as they regulate multiple biological functions, including maintaining the structure of cancer cell membranes and transmitting oncogenic signals (23–25). Increased fatty acid metabolism not only maintains the rapid proliferation rate of cancer cells but also provides them with the necessary energy source under metabolic stress conditions to maintain uncontrolled growth and improved survival of cancer cells (26). In this study, an urethane-induced lung cancer mice model was used, and the role of anthocyanins in lung tumorigenesis was elucidated by in vivo and in vitro experiments. The results showed that 0.5% ACN diet inhibited the development of urethane-induced lung cancer in C57BL/6J mice, and the mechanism might be through modulating of AMPK/mTOR signaling pathway to regulate tumor fatty acid and energy metabolism.

## Materials and methods

### Animals and experimental design

Wild-type C57BL/6J male mice were purchased from and kept in the laboratory animal facility of Chongqing Medical University. All animal care and experimental procedures were reviewed and approved by the Animal Care and Use Committee at Chongqing Medical University (IACUC-CQMU-2023-0081), and all procedures were performed in accordance with the relevant guidelines and regulations.

**Fig. 1:**
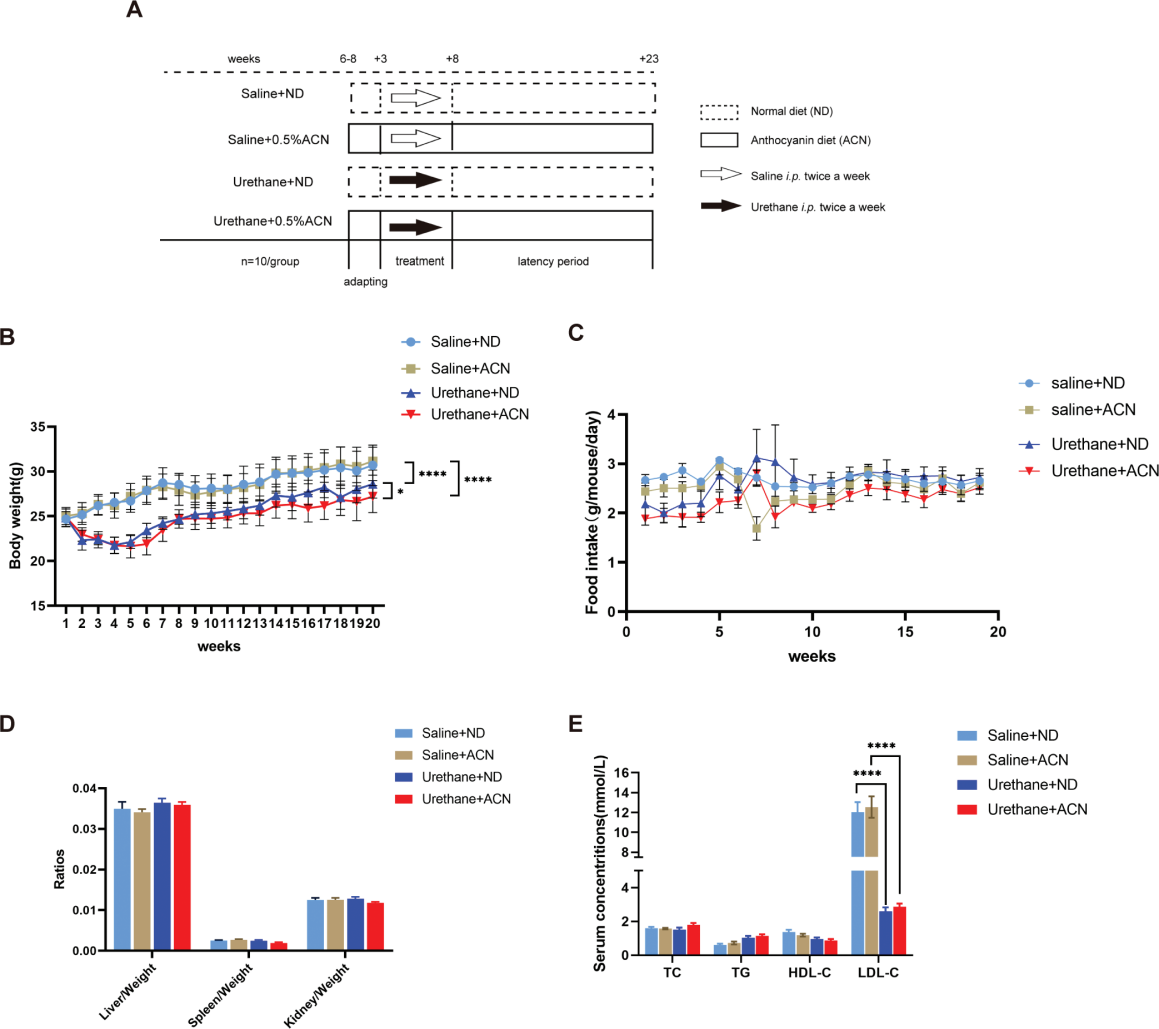
The effects of ACN on C57BL/6J mice’s body weight, organ ratio, and serum lipids. (a) Schematic diagram of the experimental design for multiple urethane injections-induced lung carcinogenesis model in C57BL/6J mice. (b) Body weight growth rate curves for ND- and ACN-fed mice. Body weights of mice measured once a week. (c) Daily intake of food according to weekly measurement. (d) Organ ratios of mice in each group. (e) Levels of total TC, TG, LDL cholesterol, and HDL in serum. *n* = 9–10 mice/group. Data are presented as mean ± SEM. *P < 0.05, ****P < 0.0001.

Six-to eight-week-old male, wild-type C57BL/6J mice were obtained from Beijing Vital River Laboratory Animal Technology Co., Ltd. and housed in an Individual ventilated caging system (constant temperature and humidity,12 h light/dark cycle, and ad Librium access to sterile food and water). Forty mice were randomly divided into two groups, fed with either normal diet (ND) or anthocyanin diet (0.5% ACN). The formula for the diets is presented in supplementary Table S1. After 3 weeks of adaptation to the feeding conditions, mice were further divided randomly into the experimental and control groups. All mice in the experimental group received an intraperitoneal injection of urethane (1 mg/g body weight, Sigma-Aldrich Inc., USA) twice a week for 5 consecutive weeks, whereas the control group received the same volume of saline with intraperitoneal injection. Lung tumorigenesis was assessed after a 15-week latency period (Figure 1A).

### Serum collection and parameter analyses

The mice were fasted for 12h before sample collection. Prior to euthanasia, 1% sodium pentobarbital was injected intraperitoneally at a concentration of 0.1 ml/10 g body weight. Then, cardiac blood was collected using a 1 mL syringe. After storage for 2–3 h at room temperature, the blood was centrifuged at 400 × g for 15 min, and the serum was collected and stored at −80°C before use. Serum parameters, including total cholesterol (TC), triglyceride (TG), and high /low density lipoprotein cholesterol (HDL-C and LDL-C), were determined using commercial kits (Nanjing JianCheng, Nanjing, China), according to the manufacturer’s instructions.

**Table 1:** Dietary composition and energy content of the experimental diets.

Ingredient (%)	ND	ACN
Casein,80 Mesh	20	20
L-Cystine	0.3	0.3
Com Starch	39.7	39.7
Maltodextrin 10	13.2	13.2
Sucrose	10	10
Cellulose, BW200	5	5
Soybean Oil	7	7
Complex mineral	3.5	3.5
Multivitamin	1	1
Choline Bitartrate	0.25	0.25
Pigment	0.05	0.05
Anthocyanin	0	0.5
**Energy (%)**		
Protein	20	20
Carbohydrate	67.9	67.9
Fat	7	7
Ingredient (%)	ND	ACN
Casein,80 Mesh	20	20
L-Cystine	0.3	0.3

### Lung tumor enumeration and histology assay

After the mice were euthanized, the trachea was exposed. With tracheal intubation, the lung tissue was expanded slowly by injecting approximately 2–3 mL of 4% paraformaldehyde. The surface lung nodules were carefully counted under a dissecting microscope by two experimental researchers who were blinded arranged (27). The lung tissue specimens were fixed with 4% paraformaldehyde, embedded with liquid paraffin, and sectionalized with a 4 µm thickness for hematoxylin and eosin (H&E) staining. The slices were roasted overnight at 60°C, dewaxed in xylene, fully hydrated with gradient alcohol at a concentration of 100–95–80–75%, washed with distilled water three times, soaked in hematoxylin dye for 3–5 min, rinsed with tap water, observed for the degree of nuclear staining under a microscope, adjusted for the depth of nuclear staining with 1% hydrochloric acid ethanol, and stained with an eosin dye solution for 7–10 min at room temperature. Finally, the slices were dehydrated and sealed with a neutral resin. Images were captured with the digital biopsy scanner (Pannoramic DESK; Hungary).

### Transcriptome analysis

Total RNA was extracted using the TRIzol reagent (Invitrogen, CA, USA), according to the manufacturer’s protocol. RNA purity and quantification were evaluated using the NanoDrop 2000 spectrophotometer (Thermo Scientific, USA). RNA integrity was assessed using the Agilent 2100 Bioanalyzer (Agilent Technologies, Santa Clara, CA, USA). Then, the libraries were constructed using VAHTS Universal V6 RNA-seq Library Prep Kit, according to the manufacturer’s instructions. The transcriptome sequencing and analysis were conducted by OE Biotech Co., Ltd. (Shanghai, China).

The libraries were sequenced on an llumina Novaseq 6000 platform, and 150 bp paired-end reads were generated. All raw data for each sample were generated. Raw reads of fastq format were firstly processed using fastp, and the low-quality reads were removed to obtain the clean reads (28). Then, all clean reads for each sample were retained for subsequent analyses. The clean reads were mapped to the mouse genome using HISAT2 (29). FPKM of each gene was calculated, and the read counts of each gene were obtained by HTSeq-count (30, 31). PCA analysis was performed using R (v 3.2.0) to evaluate the biological duplication of samples. Differential expression analysis was performed using the DESeq2 (32). *P* < 0.05 and foldchange > 2 or foldchange < 0.5 was set as the threshold for significantly differential expression gene (DEG). Based on the hypergeometric distribution, Kyoto Encyclopedia of Genes and Genomes (KEGG) pathway enrichment analysis of DEGs was performed to screen the significant enriched term using R (v 3.2.0) (33). R (v 3.2.0) was used to draw the column diagram, the chord diagram, and bubble diagram of the significant enrichment term. Gene Set Enrichment Analysis (GSEA) was performed using GSEA software (34). The analysis was used a predefined gene set, and the genes were ranked according to the degree of differential expression in the two types of samples. Then, it is tested whether the predefined gene set was enriched at the top or bottom of the ranking list.

### Cell lines and cell culture

The adenocarcinoma human alveolar basal epithelial cell line A549 was purchased from the American Type Culture Collection (ATCC), the human large cell lung cancer cell line NCI-H460 (H460) was purchased from Stem Cell Bank, Chinese Academy of Sciences (Shanghai, China), and the human non-small cell lung cancer cell line NCI-H1299 (H1299) (CTCC-001-0032) was purchased from Zhejiang Meisen Cell Technology Co., Ltd. The A549, H1299, and H460 cell lines were maintained in Roswell Park Memorial Institute (RPMI)-1640 medium (Dalian Meilun Biotechnology Co., Ltd.) supplemented with 10% fetal bovine serum (Biological Industries, Israel) and 100 U/mL penicillin and streptomycin (HyClone, Utah, USA) at 37°C in a 5% CO_2_ incubator.

### Cell viability assay

The A549, H1299, and H460 cells were cultured in RPMI-1640 medium supplemented with 10% fetal bovine serum. The effect of Cyanidin-3-O-glucoside chloride (C3G) (MB6905, Dalian Meilun Biotechnology Co., Ltd.) on the growth and proliferation of A549, H1299, and H460 cells was measured using the 3-(4,5)-dimethylthiahiazo (-z-y1)-3,5-di-phenytetrazoliumromide (MTT) method and repeated three times. A549, H1299, and H460 cells were seeded in 96-well plates at a density of 10^4^ cells/well and 100 μL/well with RPMI-1640 medium containing 10% FBS. After seeding for 24 h, the medium was removed and replaced with 100 μL of fresh medium containing serial concentrations of C3G (25, 50, 75, 100, 150, and 200 μM). After 48 h of incubation, the cells were treated with 100 μL of fresh medium containing 10 μL 0.5% MTT solution (M1020, Beijing Solarbio Science & Technology Co., Ltd) and incubated at 37 °C for 4 h. The solution was removed, and about 110 μL of DMSO (Beijing Solarbio Science & Technology Co., Ltd.) was added to each well to dissolve the purple formazan crystals. Absorbance was determined at 490 nm by using a microplate reader (Bio Tek, USA). Results were expressed as the percentage of MTT reduction relative to the absorbance of control cells.

### Colony formation assay

Colony formation assay was carried out to evaluate the role of C3G in the proliferative potential of A549. Briefly, A549 cells with a density of 500 cells/well were seeded in six-well plates and cultured at 37°C with 5% CO_2_. After seeding for 24 h, the cells were treated with different concentrations of C3G for 48 hours. The medium was replaced with fresh culture medium every 2–3 days for 2 weeks. Subsequently, cells were fixed with 4% paraformaldehyde for 20 min and stained using 10% crystal violet for 30 min.

### EdU assay

EdU assay was conducted to assess the role of C3G in the proliferation of A549 cells. A549 cell proliferation ability was detected by BeyoClick™ EdU Cell Proliferation Kit with Alexa Fluor 488 (BeyoClick, Shanghai, China). A549 cells were seeded on 24-well plates at a density of 2×10^4^ cells/well, placed into slides, and treated with different concentrations of C3G intervention for 48 h. After that, cells were incubated with 10 μM EdU working solution for 2 h and then fixed them with 4% paraformaldehyde and permeabilized them with 0.3% Triton X-100. 0.5 mL click reaction solution was added to each slide and then stained with Hoechst. Fluorescence images were photographed under a Leica confocal microscope (Leica TCS SP2, Leica, Weztlar, Germany).

### Cell apoptosis assay

A549 cells were seeded on 6-well plates and treated with different concentrations of C3G for 48 h. Adherent cells were collected for apoptosis analysis after intervention. 5 µL Annexin V-FITC and 10 µL PI solution were provided by Annexin V-FITC Apoptosis Detection Kit (Elabscience, China) to stain the cells. CytoFLEX flow cytometry from Beckman Coulter, USA, was used to detect apoptosis. CytExpert V2.3.0.84 software (Beckman Coulter, USA) was used to analyze apoptosis rates.

### Bodipy stain

A549 cells in logarithmic growth phase were inoculated into 24-well plates containing cell slides, cultured in an incubator and treated with 100 μM C3G for 12 h. After treatment, the medium was discarded, washed once with PBS, Then fix with 4% paraformaldehyde for 20 min, discard paraformaldehyde and wash with PBS for 3 times, add 500ul Bodipy (BODIPY™ 493/503,D3922, Thermo Fisher Scientific Inc.) working solution (10μM Bodipy: PBS = 1:1000) to each well for staining for 30 min in the dark, then wash with PBS for 3 times, stain the nucleus with DAPI staining solution (AR1177, Boster Biological Technology co. Itd,) for 10 min, wash with PBS for 3 times, mount with anti-fluorescence quencher, and observe under an inverted fluorescence microscope (Leica TCS SP2, Leica, Weztlar, Germany).

### Quantitative real-time PCR

Trizol reagent (DP424, Tiangen) was used to extract total RNA of tissues, followed by reverse transcription (RR047A, Takara) with 1000 ng total RNA. qRT-PCR was performed using the SYBR Green qPCR master mix (HY-K053, MCE). Relative mRNA expression fold-change was calculated based on endogenous β-actin. Specific primer sequences (5’-3’) are shown in Table 2.

**Table 2:** Primers used in the study.

Gene	Sequences (5’ to 3’)
M: Adipoq	Forward TGTTCCTCTTAATCCTGCCCA Revers CCAACCTGCACAAGTTCCCTT
M: Fabp4	Forward AAGGTGAAGAGCATCATAACCCT Revers TCACGCCTTTCATAACACATTCC
M: Cidec	Forward ATGGACTACGCCATGAAGTCT Revers CGGTGCTAACACGACAGGG
M: Aqp7	Forward AATATGGTGCGAGAGTTTCTGG Revers ACCCAAGTTGACACCGAGATA
M: Tshb	Forward AAGCAGCATCCTTTTGTATTCCC Revers CCTGGTATTTCCACCGTTCTG
M: Ace3	Forward GAGCCTGACCTCCAAGAAATCAT Revers CTCCAGCGTGTCAGATTCATACT
M: Edn1	Forward TTCTCTCTGCTGTTCGTGACTTT Revers GGAGTGTTGACCCAGATGATGTC
H: Fasn	Forward AAGGACCTGTCTAGGTTTGATGC Revers TGGCTTCATAGGTGACTTCCA
H: Srebp1	Forward CCTGGGCACTTACAGGAAGG Revers GGTCCGATTCGTCGTCAAATAAC
H: Acc	Forward TATGGAAGTCGCCTGTGGAAATT Revers GTGGTCCCTGTTTGTCTCCATAT

### Western Blot

The treated A549 cell was harvested, and the whole proteins were extracted using a mixture of 100 µl lysis buffer, 1 µL protease inhibitor (100×), 1 µl phosphatase inhibitors (100×), and 1 µL phenylmethanesulfonyl fluoride (100×). The protein concentration was determined using the bicinchoninic acid (BCA) method. Proteins were separated using sodium dodecyl sulfate polyacrylamide gel electrophoresis (Bio-Rad, Hercules, CA, USA) and transferred to polyvinylidene fluoride (PVDF) membranes. Membranes were blocked in 5% non-fat milk in TBST for 1 h at 37°C and probed with primary antibodies overnight at 4°C. After three washes, the membranes were incubated with the corresponding secondary antibodies for 1 h at 37°C. Signals were detected with ECL western blot detection reagents (BG0015, Bioground Biotech Co., Ltd, China) using a direct chemiluminescent detection system (GelView 5000Plus, Guangzhou Boluteng Instrument Co., Ltd, China). The primary antibody in this study was anti-mTOR (1:1000, 2972s, CST), anti-p-mTOR (1:1000, 2971s, CST), AMPK (1:1000, 10929-2-AP, Proteintech), p-AMPK (1:1000, AF3423, Affinity), PPARα (1:1000, 66826-1-Ig, Proteintech), S6K (1:1000, ER31205, Huaan), p-S6K (1:1000, HA721803, Huaan), SERBP1 (1:1000, HA500210, Huaan), ACC (1:1000, ET1609-77, Huaan), and p-ACC (1:1000, HA721714, Huaan).

### Statistical analysis

Data are presented as the mean ± SEM. Student’s t tests or Wilcoxon rank-sum tests were used for comparisons between two groups, and one-way analysis of variance (ANOVA) or Kruskal-Wallis H tests were used for comparisons among four groups. Statistical calculations were carried out with GraphPad Software (version 9.0) or SPSS 25.0 (SPSS, Chicago, IL, USA). *P*-values < 0.05 were considered significant. Significance levels are expressed as follows: ns, no significant, *p < 0.05, **p < 0.01, ***p < 0.001, **** *p* < 0.0001.

## Results

### The effects of ACN on body weight, organ ratio, and serum lipids of C57BL/6J mice

The body weights of the mice in the experiment were measured once a week for 20 weeks. In mice with urethane-induced lung cancer, there was a significant difference in body weight between mice on the control diet and mice on the anthocyanin diet, but daily food intake was not different statistically significant (Fig. 1b and c). Mice treated with urethane had statistically lower body weights than mice treated with saline regardless of diet, and the difference was statistically significant. At the end of the experiment, the lung, liver, kidney, spleen, and other tissues of mice were collected and weighed, and there was no significant difference in the results of the ratio of major organs in each group (Fig. 1d). The determination of TC, TG, LDL-C, and HDL-C in serum showed that there were significant differences in LDL content between the modeling group and the non-modeling group (Fig. 1e).

### Anthocyanidin diet inhibits lung tumorigenesis in urethane-induced lung cancer in C57BL/6J mice

**Fig. 2:**
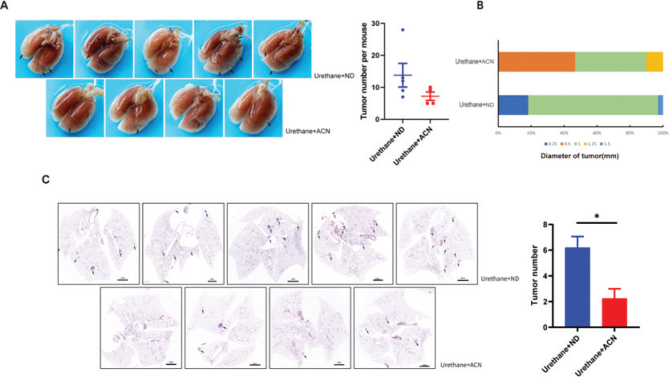
Effect of dietary anthocyanin feeding on urethane-induced lung cancer model in C57BL/6J mice. (a) Gross photographs of surface lung nodules in ND and ACN diet-fed mice. Arrows point to selective visible lung tumors. Right panel shows lung nodules per mouse. (b) Distribution of nodule diameters between two diet groups. (c) HE staining of lung sections in urethane-treated mice and number of lung tumor lesions in two diets (ND group: *n* = 5, ACN group: *n* = 4).

At the end of the experiment, the lung tissues of mice were taken and the number and size of tumor nodules were counted (Figure 2A). There was no significant difference in the total number of tumor nodules between the control diet group and the anthocyanin diet group. However, it was observed that the number of nodules > 0.5 mm in diameter in the control diet group was more than that in the anthocyanin diet group, and the proportion of mice containing larger nodules was also higher in the control diet group (Fig. 2b). Furthermore, HE staining of lung tissues revealed that the number of lung tumor lesions in the normal diet group was statistically significantly greater than that in the anthocyanin diet group (Fig. 2c), indicating that the anthocyanin diet inhibited the development of lung tumors induced by urethane in mice.

### Anthocyanin diet exerts antitumor effects against urethane-induced lung cancer in C57BL/6J mice by affecting tumor energy metabolism

**Fig. 3:**
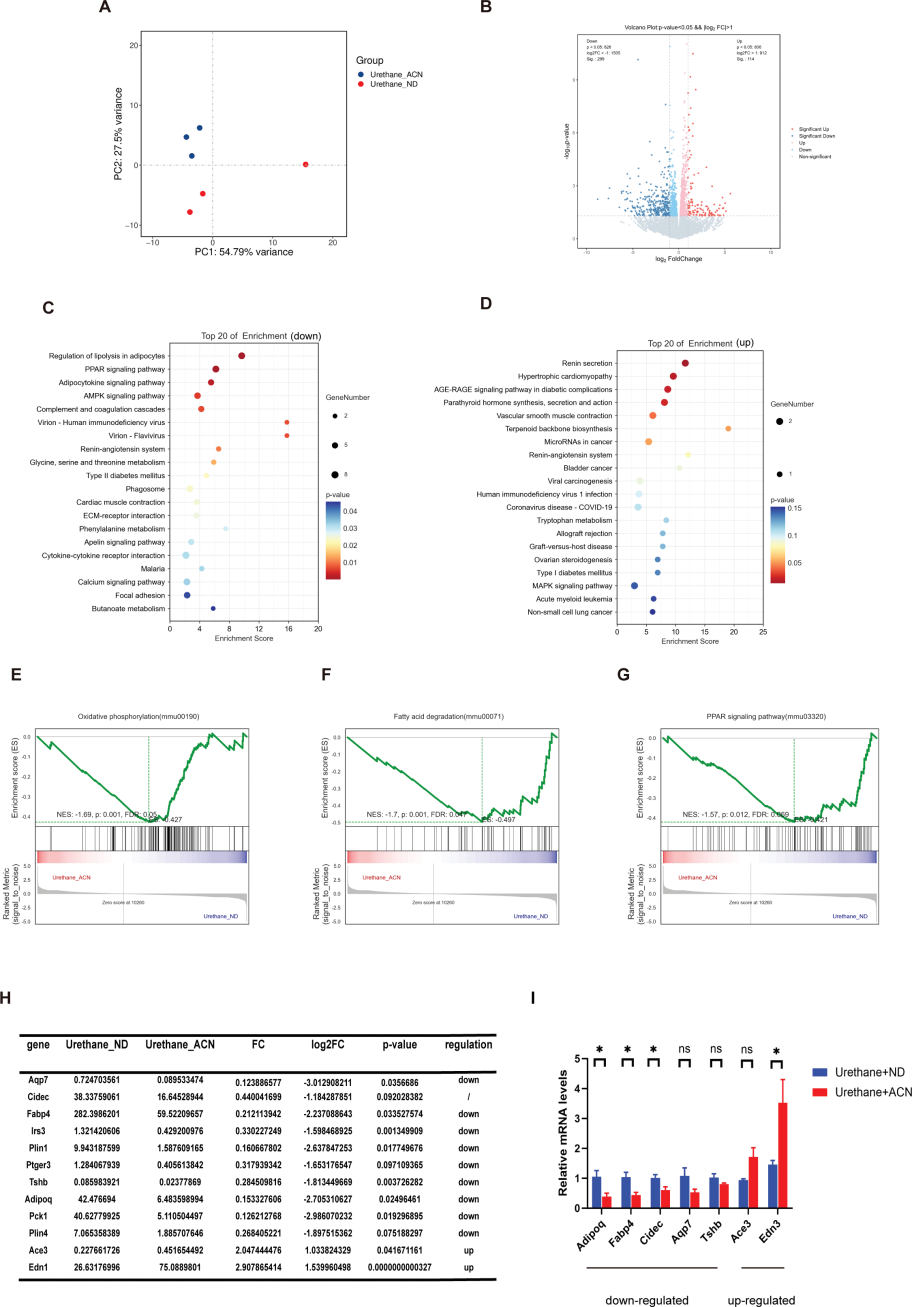
Transcriptome analysis of lung tissues from Anthocyanin or control diet. (a) Principal component analysis (PCA) score plots of the whole transcriptomic dataset in the lung. Each dot represents an observation (animal) projected onto the first (horizontal axis) and second (vertical axis) PCA variables. (b) Volcano plot of differentially expressed genes in the lung of ND and ACN group mice. Colors indicate *P* < 0.05 and fold change > 1 (red), *P* < 0.05 and fold change < 1 (blue), and non-significant (gray). (c, d) Top 20 Signaling Pathways in KEGG Enrichment Analysis. (C) The downregulated differential gene-enriched pathway, and (D) the upregulated differential gene-enriched pathway. (e–g) GSFA analysis of the entire gene set. (h) Validation of selected gene expression in the lung by RT-qPCR assay. (i) Top genes significantly expressed in down- and upregulated pathways. *n* = 3–5 mice/group, the results are presented as the mean ± SEM, representative of three independent experiments, *P < 0.05, ns, no significant.

In order to explore the mechanism by which an anthocyanin diet enhances antitumor effects, we performed RAN-seq analysis of lung tissue from urethane treated mice fed a control diet or anthocyanin diet. Principal component analysis (PCA) of the transcriptome revealed that there were significant differences in gene expression in the lung of mice with different dietary patterns (Fig. 3a). We recognized 114 upregulated and 299 downregulated genes (Fig. 3b). KEGG enrichment analysis showed that downregulated pathways were enriched in regulating lipolysis as well as Peroxisome proliferator activated receptor (PPAR) signaling pathways in adipocytes, and upregulated pathways were enriched in renin secretion and hypertrophic cardiomyopathy (Fig. 3c, d). GSEA analysis and heatmap showed significant downregulation of oxidative phosphorylation, fatty acid degradation, and PPAR signaling pathway (Fig. 3e–g). We screened some genes with significant expression from the down- and up-regulated pathways and validated them by RT-PCR, consistent with RNA sequencing, and the related genes were significantly down- and upregulated in mice fed the anthocyanin diet (Fig. 3h, i). These results suggested that anthocyanin diet might affect urethane induced lung cancer mice by altering energy metabolism in mice.

### C3G inhibits the viability, proliferation and promotes the apoptosis of lung cancer cells

**Fig. 4:**
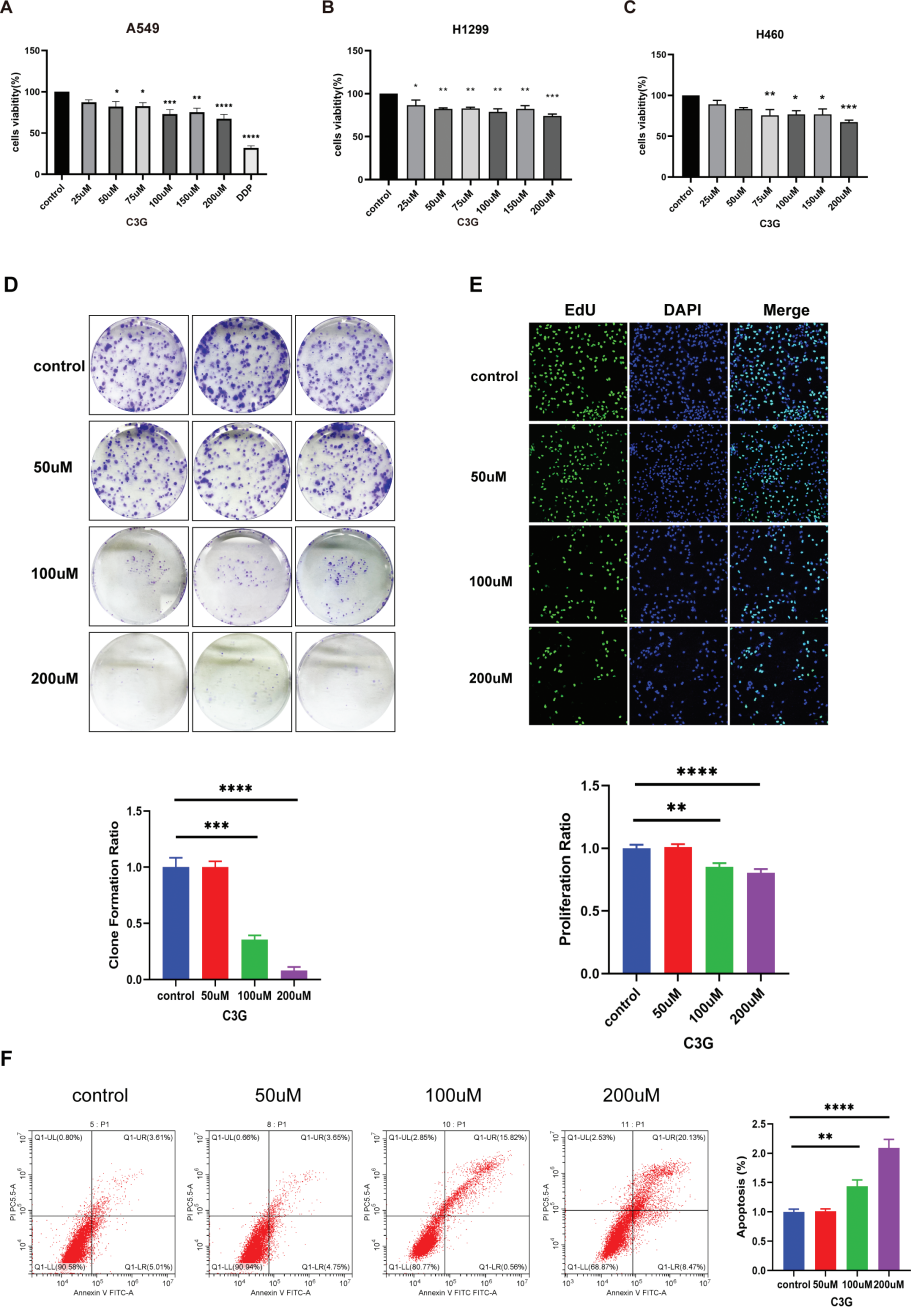
C3G inhibits the proliferation and promotes the apoptosis in lung cancer cells. (a–c) A549, H1299, and H460 cells were treated with different concentrations of C3G for 48 h. The cell viability of various lung cancer cells at different concentrations of C3G was detected by MTT assay. (d) A549 cells were treated with different concentrations of C3G, and colony formation was assessed by staining with crystal violet. (e) The percentage of EdU positive cells of A549 cells incubated with C3G was examined by EdU assay. (f) Apoptosis induced by distinct treatments for 48 h in A549 cells as illustrated by representative images. Results are the mean ± SEM. *P < 0.05, **P < 0.01, ***P < 0.001.

The effect of C3G on the viability of H1299, A549, and H460 cells was determined and assessed by using an MTT assay. H1299 was most sensitive to the intervention of C3G, followed by A549 and H460, which significantly decreased the cell viability at 25, 50, and 75 μM. In general, the cell viability of A549, H1299, and H460 cells treated with different concentrations of C3G was significantly reduced in a dose-dependent manner (Fig 4a–c). These results suggest that C3G may inhibit the growth of lung cancer cells. In subsequent experiments, C3G (50, 100, and 200 μM) was chosen to further explore the effect of C3G on A549 cells. The results of colony formation assay (Fig. 4d) and EdU assay (Fig. 4e) showed that C3G (100 and 200 μM) notably inhibited the proliferation of A549. When compared with Control group, C3G (100 and 200 μM) markedly facilitated the apoptosis of A549 cell (Fig. 4f). Altogether, the data demonstrated that C3G could inhibit the proliferation and migration and promotes the apoptosis in A549 cells.

### C3G regulates cellular fatty acid metabolism and reduces the accumulation of lipids by activating the AMPK/mTOR signaling pathway

**Fig. 5:**
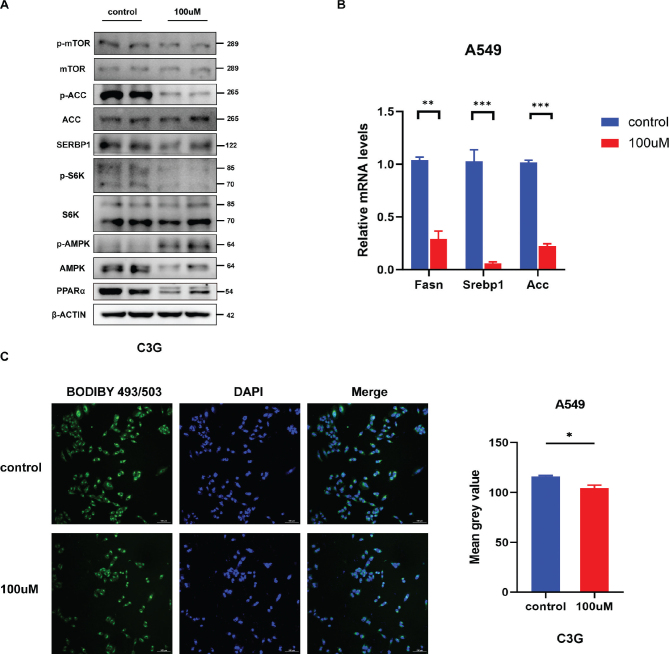
C3G regulates cellular fatty acid metabolism and reduces the accumulation of lipids by activating the AMPK/mTOR signaling pathway. (a) Western blotting for the expression of mTOR, ACC, SREBP1, S6K, and AMPK in A549 lung cancer cells. (b) Relative mRNA levels of Fasn, Srebp1, and Acc in A549 lung cancer cells. (c) Bodipy staining and staining results of A549 lung cancer cells. Results are the mean ± SEM. *P < 0.05, **P < 0.01, ***P < 0.001.

In A549 lung cancer cells treated with 100 μM C3G, AMPK/mTOR, an important energy metabolism-related pathway, was significantly altered, AMPK was activated, and its downstream target gene mTOR was significantly downregulated (Fig. 5a). As a result, the expression of fatty acid metabolism-related gene, like Fasn, Srebp1 etc. were significantly down-regulated (Figure 5B). Western blot results showed that the protein expression patterns were consistent (Figure 5A). Bodipy staining showed that the mean fluorescence intensity in lung adenocarcinoma cells was attenuated after treatment with 100 μM C3G, indicating that lipid accumulation was reduced (Fig. 5c). These results suggest that C3G may play an anti-lung cancer role by activating the AMPK/mTOR signaling pathway to affect fatty acid metabolism and reduce lipid accumulation, thereby affecting the energy metabolism of cells.

## Discussion

The mechanism of anthocyanin’s anticancer activity, particularly elucidated by in vivo models of lung cancer, has not been extensively investigated previously. We found in this study that feeding mice a diet containing 0.5% anthocyanin prevented the growth of tumors, as shown by a reduction in tumor volume and tumor number; Transcriptome sequencing analysis of mouse lung tissue and in vitro results showed that anthocyanins may inhibit tumor growth by affecting tumor energy metabolism. In vitro, C3G intervention was found to significantly inhibit the proliferation and apoptosis of lung cancer cells by regulating fatty acid metabolism through modulating the AMPK/mTOR signaling pathway.

In previous studies, anthocyanin-rich diets were proven to be able to reduce body weight and combat high-fat diet-induced obesity (35). While, the weight loss after anthocyanin diet administration was only observed in mice treated with urethane over a period of 20 weeks, but not in the saline group. To meet increased bioenergetic and biosynthetic demands and to alleviate the oxidative stress required for cancer cell proliferation and survival, cancer cells modify their flux through various metabolic pathways autonomously (36). We postulate that the decrease in body weight in mice with lung cancer is associated with tumor-specific energy metabolism, based on the sequencing results of the latter transcriptome. We also observed a significant decrease in serum LDL-C levels in a urethane-induced lung cancer mouse model, which may be associated with peculiarities of energy metabolism balance in the cancer state.

Subcutaneous transplantation lung cancer model was widely used to study the anticancer effect of anthocyanins in vivo previouly. Chen PN et al. transplanted LLC cells into C57BL/6 mice treated with saline or black rice extract and found that tumor growth was inhibited in mice in the later group (18). Kausar H et al found that both the native mixture of anthocyanidins from bilberry and the most potent anthocyanidin, delphinidin significantly inhibited the growth of H1299 xenografts in nude mice (5, 37) Recently, studies have used C3G combined with 5-FU to treat large cell lung cancer in nude mice, and found that mice treated with C3G alone or both C3G and 5-FU showed impaired tumor growth relative to control mice (38). While, due to the limitation of subcutaneous transplantation cancer model, the mechanism underlied could hardly be explored extensively. Still, the antitumor mechanism of anthocyanins was studied in vitro. It’s found that C3G could inhibit the proliferation, migration and invasion, and also facilitate the apoptosis through downregulating TP53I3 and inhibiting PI3K/AKT/mTOR pathway in H1299 and A549 cells (39). Delphinidin reduces cell proliferation and induces apoptosis of non-small-cell lung cancer cells by targeting EGFR/VEGFR2 signaling pathways (40). In our study, the antitumor effect and mechanisms of anthocyanin treatment were both investigated in vivo and in vitro. The AMP-activated protein kinase (AMPK) signaling pathway was significantly down-regulated after anthocyanin treatment. AMPK is a major regulator of energy homeostasis in cells and organisms, which coordinates multiple metabolic pathways to balance energy supply and demand, and ultimately regulates cell and organ growth (41). Mammalian target of rapamycin (mTOR) is an evolutionarily conserved serine/threonine protein kinase that regulates a variety of cellular processes, such as cell growth, cell cycle, cell survival, and autophagy (42). mTOR is one of the downstream targets of AMPK; studies have revealed that AMPK activation limits mTOR activity, and that they work together to control energy balance (43). Therefore, the observed inhibition of lung cancer by anthocyanins may be related to tumor energy metabolism.

Cancer is fundamentally a disorder of cell growth and proliferation that requires various nutrients, such as glucose, protein, and lipids, to support growth and proliferation. In recent years, fatty acid metabolism in cancer cells has received increasing attention. Because fatty acids are important for cell proliferation, inhibiting their metabolism is likely to be an effective strategy for preventing tumor growth (22). In vitro and in vivo, we observed significant alterations in genes involved in fatty acid metabolism. Acetyl-CoA catalyzes the carboxylation of acetyl-CoA to malonyl-CoA by acetyl-CoA carboxylase (ACC), which is the rate-limiting step in fatty acid synthesis, and ACC activity is regulated by AMPK, which inhibits ACC by phosphorylation, thereby blocking fatty acid synthesis (44). Fatty acid synthase (FASN) is involved in different biological processes such as plasma membrane synthesis and signal transduction, while its expression is mainly regulated at the transcriptional level by cholesterol regulatory element-binding protein (SREBP1) (45). Lipin-1 has been found to decrease SREBP transcriptional activity in the nucleus, whereas mTORC1 phosphorylation leads to inactivation and retention of Lipin-1 in the cytoplasm, thus preventing its entry into the nucleus (46). KRAS mutations in NSCLC can also enhance SREBP activity by activating the extracellular signal-regulated kinase (ERK) 1/2-mTORC1 pathway, which subsequently promotes FASN expression and increases fatty acid synthesis (24, 47). Therefore, we speculated that anthocyanins inhibit tumor cell growth by activating AMPK and inhibiting mTOR and its downstream target genes associated with fatty acid metabolism.

In vivo, we found significant downregulation of fatty acid degradation and PPAR signaling as well as inhibition of mitochondrial function. PPARs are fatty acid-activated transcription factors of the nuclear hormone receptor superfamily that are able to regulate energy metabolism (48). Taking into account whether anthocyanins affect related metabolic pathways through PPAR signaling. We selected a number of genes with significant expression from fatty acid degradation and PPAR signaling pathways and validated them by RT-PCR. Interestingly, we found that fatty acid metabolism was significantly altered. Because fatty acids are important for the proliferation of cancer cells, fatty acid utilization can be limited in several ways from the perspective of lipid metabolism, such as 1) blocking FA synthesis, 2) increasing FA degradation via oxidation, 3) diverting FAs to storage, or 4) decreasing FA release from storage (22). Catabolism of lipids occurs mainly through β-oxidation of fatty acids, which occurs mainly in mitochondria, where fatty acids are catabolized through the β-oxidation pathway, thereby increasing the catabolism of fatty acids and reducing their storage. Cancer cells can acquire lipids through the external environment. Interestingly, we also found a decrease in the expression of genes involved in exogenous fatty acid uptake (Fabp4). FABP4 belongs to fatty acid binding protein (FABP) family, which controls the metabolism and transportation of long-chain fatty acids (49). Our study showed that exogenous fat uptake genes were significantly downregulated, illustrating that anthocyanins affect their energy supply by reducing fatty acid intake in cancer cells. Cell death-inducing DFF45-like effector C (CIDEC) (also known as fat-specific protein of 27 kDa) (FSP27) localizes to the surface of lipid droplets (50). It has been found that transcripts of Cidec are upregulated by insulin, but downregulated by TNF-α, thus exerting antilipolytic and proenergy storage effects (51). In vitro, we also found that C3G intervention activated the AMPK/mTOR signaling pathway as well as downregulated PPARα expression and reduced lipid accumulation, and these results suggest that anthocyanins may play an anti-tumor role by affecting fatty acid metabolism and reducing energy supply to cells.

## Conclusions

Overall, Anthocyanin could inhibit the development and progression of urethane-induced lung tumors in C57BL/6J mice, and the mechanism was partially related to the the modulation of AMPK/mTOR signaling pathways and reduced intracellular lipid accumulation. The study provides new ideas for the possibly application of anthocyanins in the prevention of lung cancer. Still, additional mechanistic and clinical studies need to be performed.

## Authors’ contributions

H.L., M.Y.G.; Conceptualization, Methodology, Software, Writing – Original Draft. H.L., Q.Y.C.; Data curation. X.M.L.; Resources, Writing – Review & Editing, Supervision. All authors have read and agreed to the published version of the manuscript.
